# From phylogeny into ontogeny with Claes Hellerström

**DOI:** 10.3109/03009734.2016.1152332

**Published:** 2016-03-16

**Authors:** Kjell Asplund

**Affiliations:** Riksstroke, Medicine, Department of Public Health and Clinical Medicine, Umeå University, Umeå, Sweden

My research career started with snakes. For a year, from 1965 to 1966, Claes Hellerström and I worked together in herpetology (the branch of zoology that deals with amphibians, including frogs, toads, and reptiles). We joined forces with an SAS pilot, Captain Florin, who, between the flights to Los Angeles, was chairman of the Uppsala Herpetological Society. In his spare time, he collected rattlesnakes in the Californian deserts, transported them in the cockpit of his plane, and delivered them to us. Our second supplier was a herpetologist located close to Arlanda airport. Vipers on demand—as we needed snakes, he went out into the forest and collected them for us.

Why snakes? In histology textbooks from the 1950s, the pancreatic islets were described as being composed of three cell types: alpha (A), beta (B), and delta (D). It had long been known that the beta cells produce insulin. Although a substance with hyperglycemic properties—glucagon—had been detected in pancreatic extracts as early as 1923 (1), it was not until the early 1950s that it was discovered that alpha cells produce glucagon (2).

In 1960, Claes Hellerström and his PhD supervisor Bo Hellman described a silver impregnation technique that made it possible to distinguish between two types of alpha cells, which they termed A_1_ and A_2_ cells (3,4). They made the case that the A_2_ cells were the glucagon producers. The question then remained: what is the function of the A_1_ cells? This is where phylogenetic studies, including those on reptiles, came into the picture.

## The A_1_ cell enigma

Silver-positive A_1_ cells were found to be relatively uncommon in mammals; beta cells outnumbered other islet cell types by far. Hellman and Hellerström had therefore been conducting some of their early silver impregnations in pancreatic islets of chickens and ducks, where alpha cells were more abundant (3). However, even in birds only a minority of the alpha cell population was silver-positive (A_1_ cells). Was it possible they could be more prevalent in animals earlier on the evolutionary chain?

In Claes Hellerström’s PhD thesis from 1963, he included not only classical histological studies (on the two types of alpha cells) but also a method to isolate pancreatic islets from mice, a procedure that paved the way to cell physiology studies—a more progressive and successful route (5) compared to traditional histological studies.

Nevertheless, Hellerström recruited me in 1964 as one of his first two research aspirants (the other aspirant is now editor of this journal); I was to help him go back into phylogeny. Hellerström, together with his supervisor Bo Hellman, had already plunged into herpetology. In 1962, they published a paper on the pancreatic islets of bullfrogs (6). Now that Hellerström was on his own, he took on snakes and turtles. If he could find a species where A_1_ cells were particularly common, he could get a glimpse of what was the function of these mysterious cells. He could perhaps even isolate them and study their function *in vitro*.

**Figure 1.**
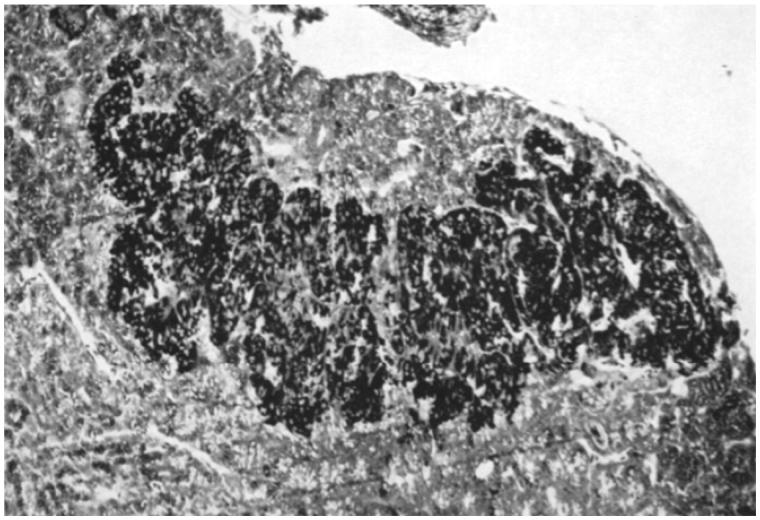
Silver impregnation (Hellman–Hellerström technique) of the pancreas of a rattlesnake. A large islet containing numerous blackened A_l_ cells is located close to the spleen. Reprinted from reference 6, Hellman and Hellerström, with permission from Springer.

**Figure 2.**
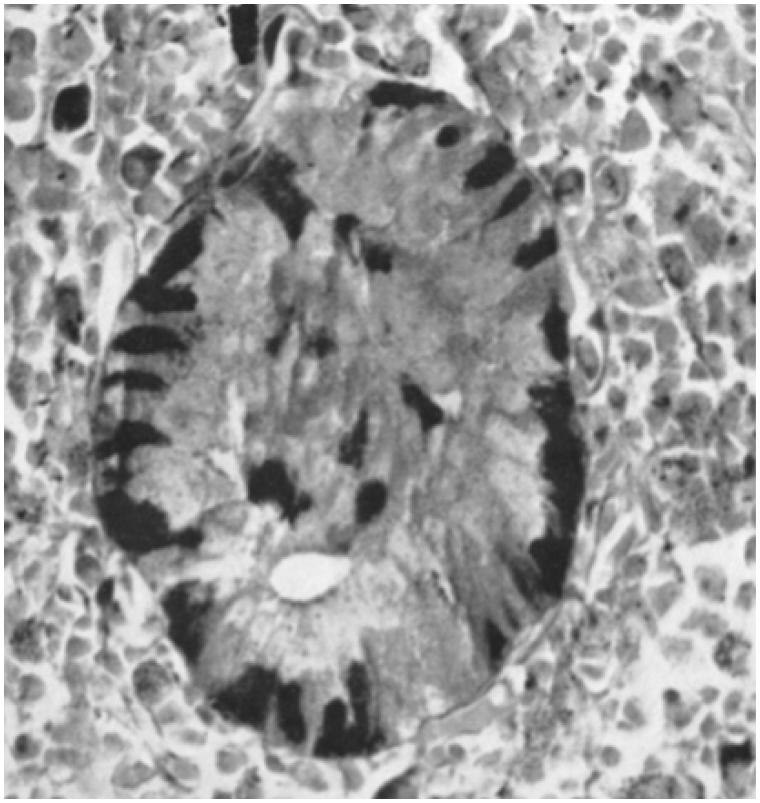
Islet from a viper imbedded in the spleen (aldehyde-fuchsin trichrome staining). The insulin-producing beta cells are stained dark. Note the lack of surrounding pancreatic acinar cells and the localization of beta cells in the periphery of the islet. ×400 in the original publication. Reprinted from reference 6, Hellman and Hellerström, with permission from Springer.

When we dissected the rattlesnakes and the vipers, we were somewhat surprised to see how easy it was to find our orientation. The location of organs was similar to that of mammals, except in a more elongated, Giacometti-like style (though I had not encountered Giacometti’s art at that time). The second remarkable observation was that we did not need a microscope to see a pancreatic islet. There was a separate, millimeter-sized organ, next to the spleen, a megastar compared to islets in other species. In the microscope, we saw islets dispersed throughout the pancreas but, as shown in Figure 1, they appeared to accumulate both in number and in size in the caudal direction, culminating in one or two extremely large islets next to the spleen (7). It seemed that this caudal movement had difficulties in stopping; in the spleen, devoid of surrounding exocrine pancreatic tissue, typical pancreatic islets were also present (Figure 2).

The hypothesis that A_1_ cells are more abundant in snakes than in mammals was thereby confirmed (Figure 1). Half of the islet cell population were alpha cells, most of which were silver-positive, i.e. A_1_ cells (7). In stark contrast to islets in mammals, only a minority of the islet cells in snakes were beta cells. Staining for granules, we found distinct granulation in both types of A cells. When we studied fresh squash preparations of microdissected islet tissue or frozen pancreatic sections in dark-field illumination, the islet cells exhibited a distinct luminescence (7). Taken together, it seemed that the A_1_ cells were producing a hormone different from insulin and glucagon. What was it?

At the time, in 1966, we simply did not have the tools available to proceed to explore the A_1_ cell enigma. Also, our enthusiasm for snakes had started to wane as an epidemic of salmonella struck the animal quarters in the basement of our institution. The culprits proved to be the rattlesnakes. Then, Captain Florin was bitten by his puff-adder, hovered between life and death, and was never able to fly again. This put a decisive end to our interest in snakes.

## Immunohistochemistry provided the answer: somatostatin

Nearly a decade passed. Histology had become an academic discipline that by the end of the 1960s seemed to have entered a scientific cul-de-sac. Claes Hellerström had arrived at this conclusion early on; he turned from cell morphology to cell physiology.

Then came immunohistochemistry. It had been conceptualized back in the 1940s. But it was not until the 1970s—when the techniques to produce a wide range of specific antibodies became more readily available—that immunohistochemistry became a frontline technique. It vitalized, even revolutionized, histology. It provided scientists with the tools to reveal the secrets of cells with unknown or unclear functions throughout the body, including A_1_ cells in pancreatic islets. Researchers at Karolinska Institute found somatostatin-containing cells in a number of endocrine tissues. In 1975, in collaboration with Claes Hellerström, they published an article in which they reported that A_1_ cells contained somatostatin (8). The A_1_ cell enigma was thereby solved. In the mid-1980s, a Canadian immunohistochemist demonstrated that somatostatin was present in the pancreatic islets of snakes as well (9).

## Reflections on our phylogeny studies

Half a century has now passed since we published our snake paper. Upon re-reading it, a few spontaneous thoughts come to mind.

First, even though both A_1_ cells and snakes turned out to be scientific dead ends, Claes Hellerström found the ideal beginner’s project for a researcher-to-be like me. The snakes were thrilling, the project was well delineated and perfectly feasible (the results were published within one-and-a-half years), Hellerström gave me a well-defined role in the project, and we made some original (although not very sustainable) observations.

Second, we published our article in *Zeitschrift für Zellforschung und Mikroskopische Anatomie*. In the mid-1960s, a journal with a grandiose German name was still prestigious. At the time our paper was published, traditional histology remained tainted by its German cultural heritage. In Uppsala, student textbooks in anatomy were in German. But *Zeitschrift für Zellforschung und Mikroskopische Anatomie* had started to adapt to the cultural shift in medical sciences and published articles not only in German but also in English (ours was in English). It survived by changing its name to *Cell and Tissue Research Journal*.

Third, it was fascinating to be part of a soon-to-be extinct species of scientists—those involved in the upfront morphology of pancreatic islets. In our 1966 article, we cited a number of papers dating all the way back to the nineteenth century. When Hellerström found out that that Vincenzo Diamare, who had published a comprehensive study on the comparative morphology of pancreatic islets in 1899 (10), was still alive, we dedicated our paper to him on his 95th birthday. Just in time, as it turned out. Diamare died later in 1966 (11). Despite the fascinating structure of snake islets and the ease with which endocrine tissue can be isolated—it can be done with the naked eye—very few original articles on snake islets have been published in recent decades (only one since the year 2000, according to the Web of Science). Our paper was last quoted in 1992.

A fourth note is that our findings on argyrophilic A_1_ cells were ephemeral. The 1975 immunocytochemistry article on somatostatin described above, with Hellerström as a co-author (8), solved a problem that had confused us when we were working with the snakes. When Hellman and Hellerström first identified silver-positive A_1_ cells, they described them as being distinctly different from D cells (3,4). We apparently confirmed this in snakes, where we found D cells to be agranular, hinting that this cell type did not produce hormone. As immunohistochemistry came along, it became apparent that A_1_ cells and D cells were identical—both contained somatostatin. Since then, our knowledge about the pancreatic islets as an endocrine organ has grown. Not only are there alpha, beta, and delta cells, but another distinct cell type that produces pancreatic polypeptide has also been added to the list (12). Whether or not there even are epsilon cells in the adult human pancreas (if so, producing ghrelin) seems to be controversial (13). But the A_1_ cells have remained unheard of since 1980 (and they do not represent the only dead end in my long publication list).

Interestingly, Lars Grimelius, an Uppsala pathologist, worked on another silver impregnation method during the 1960s. In pancreatic islets, his method apparently impregnated glucagon-producing A_2_ cells instead of A_1_ cells (14), which is somewhat of a paradox. As the Grimelius method was then found also to be useful for staining neuroendocrine tissue in other organs, it became routine in histopathology. Grimelius’s original description of the method was published in the *Upsala Journal of Medical Sciences* (at the time *Acta Societatis Medicorum Upsaliensis*) and is, by far, the most cited article ever published in this journal (15).

## Into ontogeny

During the early 1960s, Claes Hellerström’s interest in phylogeny was paralleled by his curiosity in ontogeny. Together with others in the Uppsala group, he published studies on the morphology of pancreatic islets in human fetuses (16) and in neonatal rats (17,18).

During the same period, Claes Hellerström’s interest turned from morphology to function. It is telling that the same year that our morphological paper on the pancreatic islets of snakes was published (1966), Hellerström published his first article on the metabolism of isolated mouse islets (19). He saw the limitations of traditional histology and the great potential in the emerging new cell physiology. The turn from phylogeny to ontogeny and from morphology to function was a strategic decision, which soon proved to be rewarding.

So, ontogeny and function—how to combine these new research directions? Hellerström was a certified physician and sought applications for his research that were more practical than snakes could offer. In the 1960s, pregnancy was often seen as a dreaded complication for women with diabetes. There was a considerable mortality among pregnant mothers, there was a high fetal and neonatal mortality, malformations were common, and delivery was complicated by the excessive size of the babies. Early after birth, infants born to mothers with diabetes were prone to develop hypoglycemia.

This situation raised a number of basic questions. How do insulin biosynthesis and release mechanisms mature during fetal life? Is functional maturation affected by the mother’s hyperglycemia? Could experimental studies in the offspring of pregnant rats also provide some insights into how diabetic pregnancy should be managed to avoid harm to the fetus and the newborn? This became the themes of my PhD studies under Claes Hellerström’s tutorship.

Now that I read those papers half a century later, I am less sure that Hellerström (and certainly not I) had these clinically very important questions in mind when the project started. Judging from what we wrote in our Introduction and Discussion sections, we were entirely driven by curiosity in the beginning. As the studies progressed, a shift can be discerned with more and more focus on clinical problems and clinical lessons. By the time I left Uppsala in 1972, we had found evidence that, in rats, the mechanisms for insulin biosynthesis and release normally do not mature until after birth (20–24). This would provide protection not only against excessive fetal growth in diabetic pregnancy but also against postnatal hypoglycemia in the infant. In poorly regulated diabetes during pregnancy, fetal exposure to hyperglycemia causes early maturation of the insulin biosynthesis and release mechanisms with high insulin levels in the fetus. We concluded that this would increase the risk of fetal macrosomia (and ensuing complications during delivery) and a high risk of hypoglycemia early after birth.

## Reflections on our ontogeny studies

The clinical lesson appeared to be simple: to protect against premature functional development of the pancreatic islets, strict metabolic control must be maintained during diabetic pregnancy. This is self-evident in today’s clinical practice, but such was not the case in the early 1970s. Instead, a common strategy was to advise young women with diabetes to avoid becoming pregnant. Our observations were certainly not the major contributor to the shift in the perception of how diabetic pregnancy should be managed, but they were one of many pieces of evidence that promoted a shift in the therapeutic paradigm. When I left the Department of Histology for clinical practice and research, Hellerström recruited Ulf Eriksson to study experimental diabetic pregnancy in more detail; this is described in Eriksson’s contribution to this volume (25).

A lesson for me on a more personal level concerned Claes Hellerström’s tutoring style. I was the first PhD student to graduate under his leadership. He must have had an intuitive gift for scientific leadership, maintaining a careful balance between giving the student an increasing level of independence and the need for scientific rigor. Revisiting my earliest publications, it is telling that I was the sole author of several of them. This illustrates not only the shift in publication practice that has occurred since then, but also Hellerström’s modesty and generosity as a tutor.

I did not fully realize the quality of my early scientific training under Claes Hellerström’s leadership until I turned to clinical research. In the clinic, there was an older generation of researchers whose scientific training mostly had been learning by doing. But, like me, several of my young colleagues in the university hospital had a PhD in preclinical experimental research. We clearly benefited from the elementary scientific training that we had obtained; we came to represent a new generation of clinical researchers.

A final reflection is that our work with pancreatic islets from fetal and newborn rats signified yet another foresighted shift in Claes Hellerström’s scientific focus—from normal to pathological states. In the last work I did under his tutorship, we induced intermittent hyperglycemia in pregnant rats and studied the effects on the fetuses (24). Hellerström soon went on to study an abundance of different animal models of diabetes.

## Summing up Claes Hellerström’s years of transition

Thus, the years I worked with Claes Hellerström in the 1960s and early 1970s were transitional, both in scientific paradigms and for Hellerström as a researcher. We experienced the end of a long era of German-oriented morphological research. Hellerström took a strategic decision to shift from morphological to functional studies and from traditional histology to cell biology. He also abandoned his old interest in phylogeny and focused on ontogeny. And he made a gradual transition from studying normal to pathological states. It was a privilege to accompany him on a small part of his innovative scientific journey, now some 50 years ago.

## Disclosure statement

The author report no conflicts of interest. The author alone is responsible for the content and writing of the paper.
